# Global cooling and enhanced Eocene Asian mid-latitude interior aridity

**DOI:** 10.1038/s41467-018-05415-x

**Published:** 2018-08-02

**Authors:** J. X. Li, L. P. Yue, A. P. Roberts, A. M. Hirt, F. Pan, Lin Guo, Y. Xu, R. G. Xi, Lei Guo, X. K. Qiang, C. C. Gai, Z. X. Jiang, Z. M. Sun, Q. S. Liu

**Affiliations:** 1 Research Center for Orogenic Geology, Xi’an Center of Geological Survey, China Geological Survey, Xi’an, 710054 China; 2 State Key Laboratory for Continental Dynamics, Department of Geology, Northwest University, Xi’an, 710127 China; 3 State Key Laboratory of Loess and Quaternary Geology, Institute of Earth Environment, Chinese Academy of Sciences, Xi’an, 710061 China; 4 Research School of Earth Sciences, Australian National University, Canberra, 2601 ACT Australia; 5 Institute of Geophysics, Eidgenössische Technische Hochschule Zürich, CH-8092 Zürich, Switzerland; 6 State Key Laboratory of Lithospheric Evolution, Institute of Geology and Geophysics, Chinese Academy of Sciences, Beijing, 100029 China; 7 Laboratory for Marine Geology, Qingdao National Laboratory for Marine Science and Technology, Qingdao, 266237 China; 8 College of Marine Geosciences, Ocean University of China, Qingdao, 266100 China; 9 Laboratory of Paleomagnetism, Institute of Geomechanics, Chinese Academy of Geological Sciences, Beijing, 100081 China; 10 Department of Ocean Science and Engineering, Southern University of Science and Technology, Shenzhen, 518055 China

## Abstract

Tibetan Plateau uplift has been suggested as the main driving force for mid-latitude Asian inland aridity (AIA) and for deposition of thick aeolian sequences in northern China since the Miocene. However, the relationship between earlier AIA and Tibetan Plateau mountain building is uncertain because of a lack of corresponding thick aeolian sequences with accurate age constraints. We here present results for a continuous aeolian sequence that spans the interval from >51 to 39 Ma from the eastern Xorkol Basin, Altun Shan, northeastern Tibetan Plateau. The basal age of the studied sequence postdates initial uplift of the Tibetan Plateau by several million years. Our results indicate that the local palaeoclimate was teleconnected strongly to the overall global cooling pattern, so that local enhanced aridification recorded by the studied aeolian sequence is dominantly a response to global climatic forcing rather than plateau uplift.

## Abstract (web summary)

The role Tibetan Plateau uplift played in Asian inland aridification remains unclear due to a paucity of accurately dated records. Here, the authors present a continuous aeolian sequence for the period >51–39 Ma, analysis of which indicates that aridification was driven by global climatic forcing rather than uplift.

## Introduction

Asian inland aridity (AIA) has been linked strongly with surface Tibetan Plateau uplift^1–7^. Details of late Eocene and early Miocene uplift history have been reported widely from different methods^8–10^. Recent studies trace an initial Andean-like Tibetan Plateau to the early Eocene^11–13^, but no related long AIA records are available for that time period. This hampers the understanding of any potential causality between early Tibetan Plateau uplift and AIA. Aeolian deposits, such as loess and red clay, are particularly valuable as indicators of dry land evolution because sizeable deserts are needed as sources for aeolian deposits^2,14,15^. Therefore, long (semi-) continuous aeolian deposits have been used widely to trace the history of AIA. Reworked and discrete aeolian deposits are also helpful for palaeoclimate reconstructions^16–18^.

**Fig. 1:**
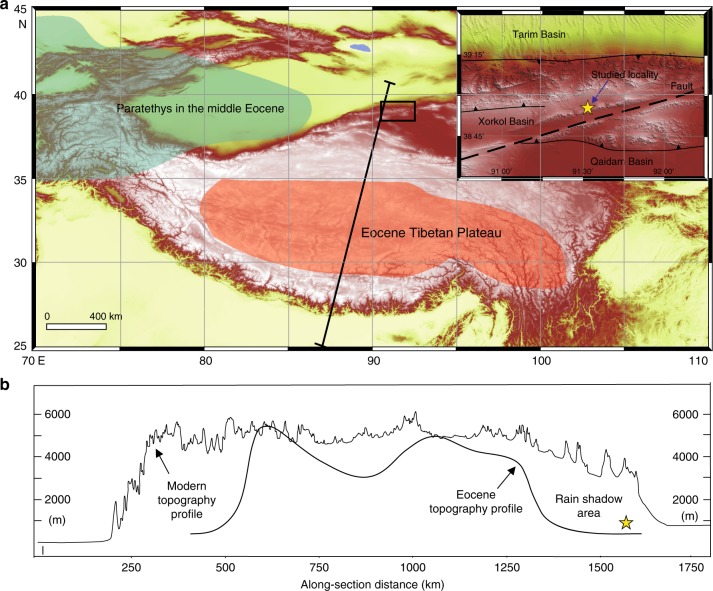
Topographic map of the study location and surrounding region. **a** Present day topography of the Tibetan Plateau and location of the study site. The sub-figure is focused on the Altun Shan and surrounding area. Also shown in **a** are the estimated extent of the Paratethys Sea^60^ and the estimated extent of the Tibetan Plateau in the Eocene^13^. **b** Topographic profiles across the Tibetan Plateau for the present day and early Eocene periods. The star indicates the location of the studied stratigraphic sequence. The early Eocene reconstruction is from ref. ^13^. The digital elevation data are from http://www.gscloud.cn/

To trace early stages of AIA development and its underlying mechanisms, we here investigated a 95.8-m red clay sequence from the Xorkol Basin (91°31′45″ E, 38°54′42″ N), which is a Cenozoic Basin within the Altun Shan, northeastern Tibetan Plateau (Fig. 1; Supplementary Figures 1 and 2; Supplementary Table 1; Supplementary Note 1). Our study demonstrates that the studied sequence spans the ~51–39 Ma time interval. The basal age of the studied sequence postdates initial uplift of the Tibetan Plateau by several million years. Our results indicate that enhanced Eocene AIA was mainly driven by global palaeoclimatic changes rather than being a direct response to the plateau uplift.

## Results

### Evidence for aeolian dust

**Fig. 2:**
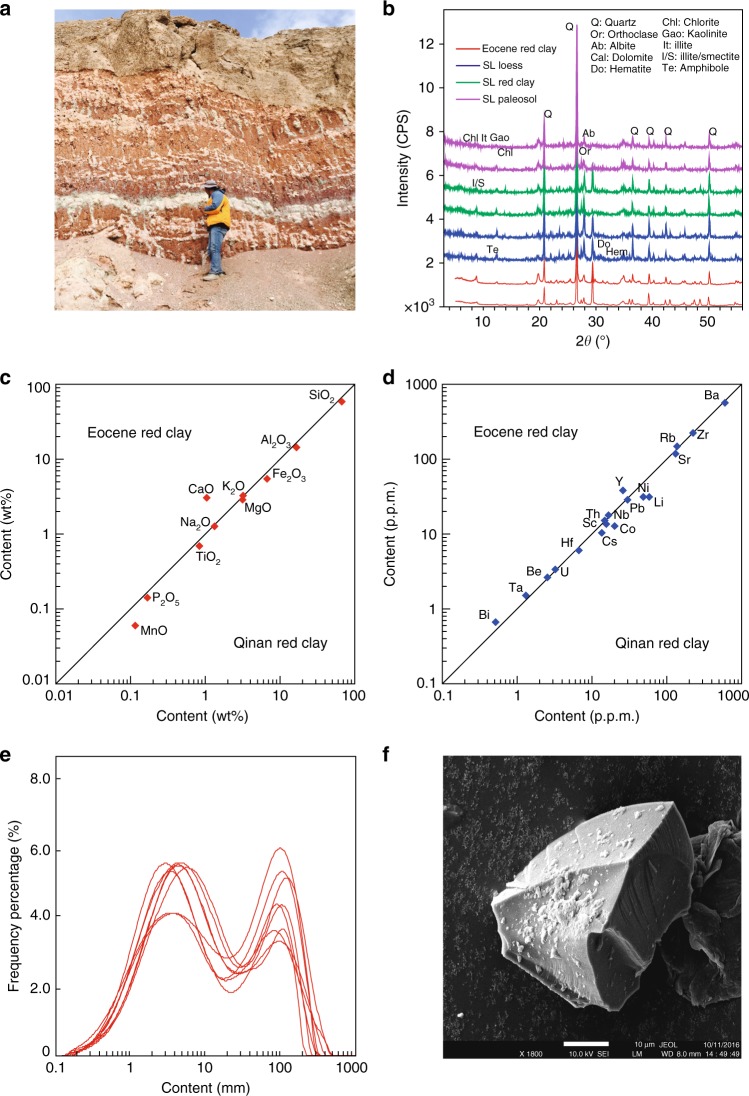
Evidence for an aeolian origin of the studied red clay. **a** Field photograph of alternating reddish-brown clay and grey caliche nodule layers. **b** Mineral composition from X-ray diffraction results from bulk sediments for the studied red clay in Altun Shan, compared with Quaternary loess and red clay from Shilou^61^ (SL) on the Chinese Loess Plateau. **c**, **d** Average major and trace element distributions for the studied red clay from Altun Shan compared with red clay from Qin’an^62^. **e** Grain size distributions for the studied red clay from Altun Shan. **f** Scanning electron microscope image of representative quartz grains that illustrate the angular morphology of the grains

Most terrestrial clastic sediments with silt-clay size particles are transported by either water or wind. Following typical studies of aeolian dust on the Chinese Loess Plateau (CLP) and surrounding areas^2,16–18^, we interpret the studied red clay sediments as aeolian deposits for the following six main reasons. First, the fine-grained sediment fraction (<100 μm) that can be transported in suspension by wind^19^ consists of up to 92% of the total sediment, which is within the typical range for aeolian dust from the CLP^20^. Second, aeolian dust on the CLP is well sorted due to long-distance transportation and accumulated at low and relatively stable rates. Chinese loess is, thus, homogenous and devoid of stratification and lamination^21^. The studied red clay also lacks bedding, but has pseudobedding (Fig. 2a, Supplementary Figure 3; Supplementary Note 2) associated with calcareous nodules derived from leaching of primary clay. Layer-cutting spindle-like calcareous nodules in some “layers” distinguish pseudobedding from periodic climatic stratification and lamination (Fig. 2a, Supplementary Figure 3). Third, mineral assemblages and geochemical features of the studied red clay are comparable to those of aeolian red clay from the CLP (Fig. 2b–d). Fourth, grain size distributions of the clastic component in the red clay have bimodal distributions (~30–100 μm and ~1–10 μm for the coarse and fine fractions, respectively) and remain relatively stable throughout the profile (Fig. 2e). The bimodal distribution is strikingly similar to Miocene-Quaternary CLP aeolian deposits that were transported by two wind systems^22,23^ and to aeolian deposits in the NE Tibetan Plateau (Fig. 2e)^16^. Fifth, quartz grains, which comprise the main granular mineral of the studied red clay, have irregular and angular shapes, with most having sharp edges (Fig. 2f), low-relief and conchoidal fracture. This is because fine particles are transported in suspension^24,25^, which decreases grain–grain and grain–bed collisions and preserves the original shape of quartz grains from the source area. Sixth, elemental signatures of the studied Eocene red clay are similar to the average composition of upper continental crust, which indicates that the sediments were derived from well-mixed sedimentary protoliths that underwent repeated upper crustal recycling (Supplementary Figure 4)^26^. The sediment must, therefore, be derived from widely mixed areas, such as Asian deserts, because local sources have more specific geochemical signatures. All of the above sedimentological and geochemical evidence attests to an aeolian origin for the studied red clay deposits (Supplementary Notes 3 and 4).

### Age model

**Fig. 3:**
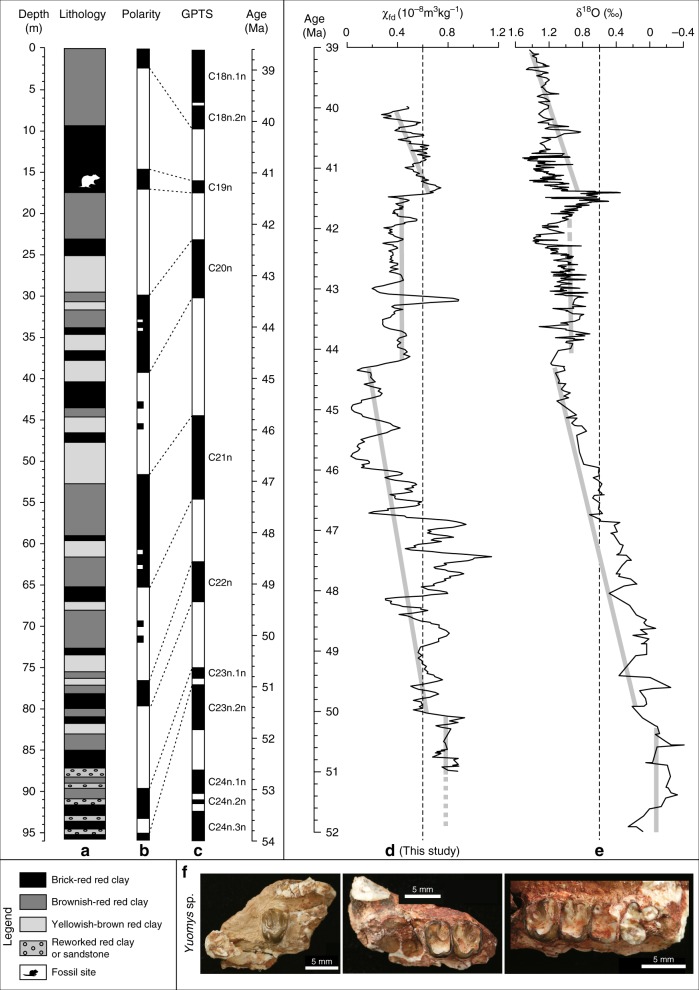
Magnetostratigraphy and frequency-dependent magnetic susceptibility for the studied red clay sequence. **a** Lithology, **b** magnetic polarity stratigraphy for the studied Xishuigou section, Altun Shan, and correlation with **c** the geomagnetic polarity timescale (GPTS)^29^. Polarity: black = normal, white = reversed. Each plotted palaeomagnetic direction was obtained by principal component analysis of detailed stepwise thermal demagnetisation data. **d**, **e** Correlation between **d** the frequency dependence of low-field magnetic susceptibility χ_fd_ of Altun Shan red clay and **e** the δ^18^O record of ref. ^32^. The χ_fd_ record reflects pedogenic formation of fine magnetic particles, which depends strongly on soil moisture. It is, therefore, a good proxy for precipitation/aridity^31^. The long-term χ_fd_ trend and recording of humid events such as MECO at ~40–41 Ma (see main text) correlate well with the global climate record of ref. ^32^ and support the age model presented. Minor discrepancies in the positions of events in **d** and **e** largely reflect imprecision in the respective age models. **f** Photographs of extinct Eocene *Yuomys altunensis* rodent jawbones are shown at the base of the figure

The red clay is overlain disconformably by the Caihonggou Formation, which was deposited between 13 and 2.6 Ma^27^, and indicates a minimum age of 13 Ma for the studied red clay. Furthermore, we discovered *Yuomys altunensis* at a depth of ~17.5 m within the studied sequence that belongs to the Sharamurunian period, which spans the late Middle Eocene age interval^28^. Constrained by the Eocene age for the *Yuomys altunensis* fossils, we correlated our newly defined palaeomagnetic polarity sequence to the geomagnetic polarity timescale^29^. The extrapolated uppermost and basal ages are ~39 Ma and ~51 Ma, respectively (Fig. 3; Supplementary Figures 5–12; Supplementary Notes 5–10).

### Correlation between χ_fd_ and δ^18^O

On the basis of the biostratigraphically tied magnetostratigraphic age model, a notable correlation between the frequency-dependent magnetic susceptibility (χ_fd_) and the global marine oxygen isotope (δ^18^O) record is observed (Fig. 3d, e). χ_fd_ is sensitive to ferrimagnetic nanoparticles, with grain size across the superparamagnetic (SP) to stable single-domain (SD) size range^30^. Generally, χ_fd_ is enhanced for (palaeo-) soils due to formation of SP + SD magnetic particles under warm/humid conditions and is used as a precipitation proxy^31^. Marine δ^18^O data provide information on ice volume variations. Continental atmospheric vapour content is linked strongly to regional rainfall. Our χ_fd_ curve mimics clearly the δ^18^O results, and records not only the long-term global Cenozoic cooling trend, but also major hyperthermal events such as the Middle Eocene Climatic Optimum (MECO) at ~41.5 Ma^32^ (Fig. 3d, e). This indicates strongly that climatic variations recorded by red clay χ_fd_ in the study area were sensitive to global palaeoclimatic fluctuations on different time scales. The age model indicates that the basal age of the red clay is >51 Ma, which is much earlier than documented previously for the oldest aeolian deposits from North China and Central Asia (<22–40 Ma)^2,16–18,33^.

## Discussion

Generally, continuous long aeolian deposition requires both sizeable source areas arid enough to allow deflation and an atmospheric circulation sufficiently energetic to carry aeolian particles from the source to the depositional sinks. For example, the long loess sequences from the Chinese Loess Plateau have been taken as indicative of mid-latitude aridity^2,33^. In addition to sizeable source areas, piedmont fluvial fans^34^, reworked fluvial sediments and dry lake beds^35–37^ could have been potential dust sources. Regardless of the exact source, deposition of such a thick aeolian sequence requires a sizable and long-lived source to provide a continuous dust supply over timescales of 10 Myr, which indicates enhanced regional aridity. Palaeomagnetic data from the Tarim and Qaidam Basins, and the surrounds suggest insignificant post-Early Cretaceous northward motion, which indicates that these areas have been at the same latitude since the Cretaceous^38–40^. Therefore, the studied Xorkol Basin aeolian sequence provides a record of mid-latitude AIA that can be traced at least to >51 Ma.

Palaeoclimatic changes (e.g., progressive global cooling in a long-term hot and dry continental environment), retreat of the Paratethys Sea and Tibetan Plateau uplift are possible factors that contributed to the documented enhanced mid-latitude AIA^1–7,9,11,13,17,18,33,41,42^ and then to long-term aeolian deposition in the Xorkol Basin. The interiors of large continents, such as Eurasia, are normally arid because of the distance to oceanic moisture sources and highly evaporative conditions. Given that the Tibetan Plateau was already elevated to altitudes >4,000 m in the Eocene^12,13^, moisture that reached inland areas during this period would have come from the Paratethys Sea to the west (Fig. 1a), over the Tibetan Plateau from the Indian Ocean to the south or from the east for a strengthened East Asian Monsoon.

Our measured χ_fd_ signal is carried by nanosized magnets that were formed during pedogenesis. Extensive studies of Chinese Loess and modern soils distributed along a precipitation gradient indicate that χ_fd_ is proportional to annual precipitation. Temperature is not evidently important for χ_fd_ changes. χ_fd_ values for the studied sequence are ~0.8 m^3^ kg^−1^ at ~50–51 Ma, which corresponds to modern annual precipitations <~300 mm per yr^43^, which strongly indicates dry regional conditions at the onset of aeolian deposition in the Xorkol Basin at ~50–51 Ma. Excellent correspondence between the marine δ^18^O record and the Altun Shan χ_fd_ record through the profile (Fig. 3d, e) provides key information on the subsequent control of AIA by climate evolution. Coupling between marine δ^18^O and magnetic susceptibility of younger loess and red clay deposits reveals that global climate was the main driver of local rainfall variations^44^. Based on the correspondence between marine δ^18^O and Altun Shan χ_fd_ variations, we conclude similarly that global cooling was the main factor that drove aeolian deposition in the Xorkol Basin. The key question is how did global cooling lead to increased aeolian deposition in this part of the Asian continental interior? Our evidence suggests strongly that global cooling after ~50–51 Ma decreased moisture availability (controlled by the precipitation/evaporation balance) to drive progressively drier conditions indicated by the decreasing χ_fd_ trend. We lack detailed records older than 51 Ma, so we cannot provide an exact mechanism for the onset of enhanced AIA in warm periods prior to ~51 Ma, but the studied region has evidently had a dry climate since the early Eocene, in which progressively decreased moisture availability was the major factor in controlling the signals recorded in the studied palaeoclimatic archive. Ongoing Cenozoic global cooling could have contributed to regional environmental changes in Asia by reducing the intensity of the global hydrological cycle and by intensifying dry conditions^45^. δ^18^O records indicate that the onset of long-term global cooling from greenhouse to icehouse conditions started from ~51 Ma^32^, which coincides with the basal age of the studied aeolian sequence, and suggests strongly that global climate cooling was an important factor for AIA.

The Paratethys Sea, which was a significant moisture source and climate regulator via changing land–sea thermal contrast, played a critical role in the climatic evolution of interior Asia^7,41^, as supported by geological evidence^3,46^. The Paratethys Sea, which was connected to the central Atlantic Ocean, extended to the Tarim Basin in the east during the early Cenozoic era^47,48^. The eastern extremity of this vast region is now occupied by the Tibet–Pamir orogenic system and related sedimentary basins, including the Tajik and Tarim Basins^46^. Recent studies from both of these basins indicate that the Paratethys Sea retreated from Central Asia not earlier than ~40 Ma, which implies that there was no clear land–sea redistribution that coincided with the accumulation of the studied aeolian sequence^3,46–48^. The low χ_fd_ values compared with those of Quaternary CLP palaeosols indicate limited precipitation in this region^43^. Nevertheless, excellent correlation between our χ_fd_ record and the global marine δ^18^O record indicates strongly that vapour transportation was connected to the global climatic system because weak moisture supply to the Altun area would have originated mainly from the Paratethys Sea since the early Eocene^11^.

Previous studies have hypothesised a link between Plateau uplift and Central Asian interior climate^1–7^. Rising vapour from the Indian Ocean cools, condenses and precipitates before it crosses the Tibetan Plateau, which results in drier air advancing to the northern Plateau. If the orographic barrier is high enough, vapour will be blocked to create a typical rain shadow effect on the lee side of the high mountains, which has been demonstrated by the building of high relief as a result of India–Asia collision since the late Eocene to early Miocene^1–7^. Ding et al.^13^ reconstructed palaeoelevations using the most negative oxygen isotope values, which suggest an elevation of about 4500 m on the southern margin of the Lhasa terrane that resulted from early Eocene subduction of the Indian plate. Before ∼50 Myr, southern Tibet had grown into a mountain belt, resembling the Central Andes, that was 200–400 km wide and perhaps ∼4000 m high, extending from roughly 10°N ( ± 5°) at ∼90°E to 20°N ( ± 5°) at ∼70°E^12^. This long-standing topographic feature was high enough and extensive enough in the early Eocene to produce a rain shadow on the lee side of the plateau, which blocked the southerly sourced moisture to produce a distinct spatial palaeo-precipitation distribution with low δ^18^O values in the rain shadow and high δ^18^O values for westerly driven precipitation in Central Asia, respectively^11^. This topographic limitation of moisture supply likely contributed to mid-latitude AIA and deposition of the studied aeolian Altun Shan sediments. However, the basal age of the studied aeolian sequence postdates initial southern Tibetan Plateau uplift by several million years. This indicates strongly a lack of causality between AIA and Tibetan Plateau uplift. In contrast, several lines of evidence indicate that Tibetan Plateau uplift should have increased hydrological activity. For example, Ruddiman and Prell^49^ and Licht et al.^16^ argued that high topography should have further increased monsoon precipitation. Nie et al.^50^ also demonstrated that East Asian summer monsoon precipitation penetrated further inland to the Qaidam Basin during the late Miocene, when different lines of evidence suggested a phase of northeastward Tibetan Plateau growth. Therefore, we conclude that plateau uplift was not the dominant driver of this AIA. We note that closed basins were present in inland Asia for much of the Mesozoic and Cenozoic eras^51,52^. Red-bed deposits with extensive dune fields and thick pedogenic/caliche carbonates indicate localised aridity before the early Cenozoic era in northern China^53–56^. Thus, although the studied aeolian sequence does not provide the oldest evidence for AIA, its basal age strongly indicates that AIA was enhanced at ~51 Ma.

Preservation of aeolian deposits is extremely sensitive to erosion. We attribute cessation of aeolian deposition (at ~39 Ma) to a change in local depositional conditions. First, the Paratethys Sea had retreated from Central Asia by ~40 Ma^3,46,47^, which implies that transportation of humid vapour into the Altun Shan was reduced from that time. Such moisture sources are needed to release, transport and deposit silt during humid periods that can then be deflated during subsequent arid periods^50,57^. Second, preservation of red clay sequences requires a relatively stable tectonic environment during and after deposition^42^. Fission track studies indicate that the Altun Shan underwent uplift during the middle-late Eocene^58,59^. Thus, we suggest that aeolian deposition ended at ~39 Ma, possibly due to a combination of reduced moisture supply and increased local tectonic instability.

The studied red clay sequence is the oldest known aeolian section from the northeastern Tibetan Plateau, which provides important insights into the regional response to global climate evolution. Specifically, the 95.8-m-thick sequence discussed here extends the known history of thick aeolian deposition in the Asian interior by ~10 Myr. Progressive global climate cooling is the most likely driver of the documented Eocene AIA. The base of our studied sequence postdated initial Tibetan Plateau uplift by several million years, and the AIA enhancement (>51 Ma) postdated initial development of the Gangdese mountains on the southern margin of the Lhasa terrane (at ~55 Ma). We conclude, therefore, that the study area was teleconnected strongly to the overall global cooling pattern, so that, local aridification recorded by the studied aeolian sequence was dominantly a response to global climatic forcing rather than plateau uplift.

## Methods

### Sampling

Oriented palaeomagnetic samples were collected in 2 ways depending on the hardness of the sediment. Discrete cubic samples (2 × 2 × 2 cm^3^) were taken from softer sediments, while cylindrical samples were drilled from harder sediments and were later cut into individual specimens (2.2 cm diameter × 2.5 cm length). Two parallel sets of samples were taken at 20–100-cm stratigraphic intervals. At each stratigraphic horizon, parallel powder samples were also taken for magnetic measurements (e.g., mass-specific magnetic susceptibility, χ) and for grain size analysis.

### Sedimentological measurements

Grain size analyses were made using a standard chemical pre-treatment procedure, in which organic material, carbonate and clay minerals were removed sequentially from the red clay. The samples were then analysed using a Mastersizer 2000 laser particle analyzer. Sample preparation, pre-treatment and analyses were done at the State Key Laboratory of Continental Dynamics, Department of Geology, Northwest University, Xi’an. Major and rare earth elements, and mineral compositions were determined at the Xi’an Institute of Geology and Mineral Resources using an inductively coupled plasma mass spectrometer (ICPMS, Thermo Elemental X Series) and a Rigaku X-ray diffractometer, respectively. Quartz micromorphology was observed with a LEO 1450VP scanning electron microscope (SEM) at the Xi’an Institute of Geology and Mineral Resources.

### Magnetic measurements

Palaeomagnetic specimens were demagnetised using an ASC TD48 oven, and palaeomagnetic directions were measured with a 2-G Enterprises cryogenic magnetometer (model 755 R) at the Institute of Geophysics, ETH Zürich, Switzerland. Both the demagnetiser and magnetometer were housed in a magnetically shielded laboratory. Thermal demagnetisation of the natural remanent magnetisation (NRM) was carried out at 25 °C or 50 °C increments from room temperature to 700 °C. Details of the magnetic properties are described in the Supplementary Material.

To determine the magnetic properties and minerals responsible for the measured palaeomagnetic signals, and to determine the best procedure for subsequent thermal demagnetisation, rock magnetic analyses were performed before palaeomagnetic analyses. Representative samples were analysed in the Palaeomagnetism and Geochronology Laboratory, Institute of Geology and Geophysics, Chinese Academy of Sciences, Beijing. Temperature-dependent magnetic susceptibility curves (χ-T) were obtained using a KLY-3 Kappabridge magnetic susceptibility meter and a CS-3 furnace with an argon atmosphere.

### Data availability

The datasets generated and/or analysed during the current study are available from the corresponding author on request.

## Electronic supplementary material

Supplementary Information
