# Supplementary material for: Global cooling and enhanced Eocene Asian mid-latitude interior aridity
Source: Nat Commun. 2018 Aug 2;9:3026. doi: 10.1038/s41467-018-05415-x (PMC6072711; doi:10.1038/s41467-018-05415-x)
Supplement: Supplementary file 1 — Supplementary Information [file 41467_2018_5415_MOESM1_ESM.pdf]

Supplementary Information

Global cooling and enhanced Eocene Asian mid-latitude interior aridity

J. X. Li[1, 2], L. P. Yue[2, 3], A. P. Roberts[4], A. M. Hirt[5], F. Pan[1,2], Lin Guo[1], Y. Xu[1], R. G. Xi[1], Lei Guo[1], X. K. Qiang[3], C. C. Gai[6], Z. X. Jiang[4,7], Z. M. Sun[8], Q. S. Liu[10*]

[1]Research Center for Orogenic Geology, Xi'an Center of Geological Survey, Geological Survey of China, Xi'an, People's Republic of China

[2]State Key Laboratory for Continental Dynamics, Department of Geology, Northwest University, Xi'an, People's Republic of China

[3]State Key Laboratory of Loess and Quaternary Geology, Institute of Earth Environment, Chinese Academy of Sciences, Xi'an, People's Republic of China

[4]Research School of Earth Sciences, Australian National University, Canberra, ACT, Australia

[5]Institute of Geophysics, Eidgenössische Technische Hochschule Zürich, Zürich CH-8092, Switzerland

[6]State Key Laboratory of Lithospheric Evolution, Institute of Geology and Geophysics, Chinese Academy of Sciences, Beijing, People's Republic of China

[7]Laboratory for Marine Geology, Qingdao National Laboratory for Marine Science and Technology, Qingdao, People's Republic of China

[8]College of Marine Geosciences, Ocean University of China, Qingdao, China

[9]Laboratory of Paleomagnetism, Institute of Geomechanics, Chinese Academy of Geological Sciences, Beijing, People's Republic of China

[10]Department of Ocean Science and Engineering, Southern University of Science and Technology, Shenzhen, People's Republic of China

[*]Corresponding author: Qingsong Liu, email: qsliu@sustc.edu.cn

Supplementary Note 1: Geological setting

The studied aeolian sequence is located in Xorkol basin **(Supplementary** Figure 1**)**, which is a Cenozoic basin in the Altun Shan. After initial regional investigations in the 1980s[1], little attention has been paid to the Cenozoic strata in this area until Yue et al. (2004, 2005)[2,3] identified large-scale extrusion tectonics based on analysis of the Tertiary basin fill and its provenance in the eastern Xorkol Basin. Recently, Miocene red clay has been studied in the eastern Xorkol Basin[4], which was previously considered part of the Palaeogene Ganchaigou Formation[1].

Delimited by high-angle reverse faults on both sides[2], Tertiary sequences of the East Xorkol Basin have been deformed into an asymmetric, open syncline with a steeper southern limb and a shallower northern limb and are overlain unconformably by the Pleistocene Qigequan Formation. According to the characteristics of the sequences and fossils within them, most of the Tertiary sediments are Miocene and Eocene red clay, respectively, with a disconformity between them. The continuously deposited Eocene red clay is only well exposed on the southern limb of the syncline, which was previously assigned as part of the Palaeogene Lulehe Formation based on correlation to the sequence in the Qaidam basin[1].

The Eocene Xishuigou Formation is 95.8 m thick, and contains about 40

visually-defined, brownish red clay layers with grey carbonate nodules. Locally, water-lain, fine-grained sandstones and siltstones contain fossils. The Xishuigou Formation is separated by a regional disconformity and is overlain by Miocene sediments, which constrains the Caihonggou Formation to be older than the Miocene. Based on the presence of *Yuomys altunensis* fossils, the red clay sequence has been assigned to the Eocene. Eocene deposits contain larger carbonate nodules than the Miocene red clay; therefore, Eocene and Miocene deposits can be distinguished readily in the field. Eocene red clay has limited outcrops due to overlap of the overlying Miocene sequence, but the presence of large red clay boulders in Quaternary deposits in adjacent areas (such as Hou Liugou) suggests that Eocene red clay must be widespread in the Xorkol basin and surrounding areas. Sedimentological and geochemical evidence attests to an aeolian origin for the studied red clay deposits (see main text). In addition, several thin sand and conglomerate layers lower in the section were evidently deposited in ephemeral aqueous environments (Supplementary Table 1, Supplementary Figure 2).

Supplementary Note 2: Bedding in mudstone and red clay

Aeolian dust is well sorted due to long-distance transportation. Accumulation at relatively stable rates results in a homogenous deposit devoid of stratification and lamination[5]. However, pedogenesis during or after accumulation contributes to the formation of leaching layers in humid environments. Eocene red clays lack the typical bedding observed for water-deposited sediments, but they contain “pseudobedding” composed of red clay and calcareous nodule layers derived from leaching of primary clay

(Supplementary Figure 3a, b). Layer-cutting spindle-like calcareous nodules in some "layers" (Supplementary Figure 3a) make it straightforward to distinguish pseudobedding from the clear stratification and lamination that is observed typically in water-lain sediments.

Supplementary Note 3: Geochemistry

The major element composition of the red clay sequence is dominated by SiO_2, Al_2O_3, Fe_2O_3, MgO, and K_2O. The rare earth element (REE) distribution pattern is characterized by light REE (LREE) enrichment, fairly flat heavy (HREE) profiles, and a distinct negative Eu anomaly. The major element composition of the studied Eocene red clay in Altun Shan varies considerably along the sequence. More importantly, major element abundances in the Eocene red clays are consistent with those of typical aeolian deposits from the CLP (both Miocene and Quaternary) (Supplementary Figure 4), which indicates that the constituent aeolian materials are well mixed during transportation and sedimentation. This is evident in the similar mineral compositions of aeolian deposits from these two far-separated areas. It is generally agreed that fine-grained aeolian material, as derived from large desert areas, is well mixed during transportation and sedimentation, and that this homogenizing effect should result in nearly constant REE distributions[6,7]. This rationale has been used widely to determine the aeolian origin of the Chinese loess[8,9], and of the red clay sequences in surrounding regions[10,11]. The REE distribution patterns of the Eocene red clay are similar to typical aeolian CLP deposits[12], with enriched LREE and relatively flat HREE profiles, and a constant negative Eu

anomaly. All measured values are also close to the average composition of upper continental crust[13], which implies that Eocene red clay shares a similar origin to aeolian CLP deposits from multiply recycled and well-mixed ancient upper crustal sediments.

Supplementary Note 4: Summary of the origin of red clay

According to Pye and Tsoar[14], aeolian deposits fall into three main categories: sand dunes, sand sheets, and loess blankets. Here, we focus on silt-size loess because it is particularly valuable as an indicator of dry land evolution and is used widely for tracing Asian interior ariditifiation (AIA)[8,10,11,15,16]. In contrast, sand dunes are not directly linked with aridity, and can be found in many settings (e.g. coastal and river bank dunes)[14], even in humid areas in south China[17]. Loess is commonly defined as an accumulation of windblown silt, which stresses the processes of material transport, and accumulation by wind[18,19]. However, thick (about 100-m) silt-clay sequences of windblown sediments can also be deposited in deep lakes and seas[20,21]. Therefore, it is necessary to distinguish loess from water-lain deposits before attempting AIA reconstructions.

Fine-grained sedimentation is usually generated by particle decantation from suspension. Deep lakes or seas with flat and extensive floors far from source areas tend to give rise to stable sedimentary settings over long time periods and support deposition of thick mudstone sequences. Horizontal bedding is typical of mudstone. Carbonate interbeds that do not cut bedding can also be important in deep lakes and marine deposits. Aeolian dust is also well sorted due to long-distance transportation, and its accumulation at relatively stable rates results in a homogenous deposit devoid of stratification or

lamination[5]. However, pedogenesis during or after accumulation contributes to the formation of leaching layers in relatively humid environments. The studied Eocene red clays lack the typical bedding observed for water-deposited sediments, but they contain “pseudobedding” composed of red clay and calcareous nodule layers derived from leaching of primary clay. Layer-cutting spindle-like calcareous nodules in some “layers” make it straightforward to distinguish pseudobedding from the clear stratification and lamination that is observed typically in water-lain sediments.

The studied Xishuigou red clay sediments are dominated by fine-grained particles (< 100 μm) that comprise up to 92% of the sediment, which means that most of the particles were transported in aeolian suspension[14,22,23]. In these transportation processes, grain-grain and grain-bed collisions are scarce and the original shape of quartz grains from the source area is well preserved. This is why sharp edges, angular morphology, low relief, and conchoidal fractures are common in sediment particles that make up loess deposits[24-26]. Thus, sedimentary characteristics indicate that the studied sediments consist of aeolian rather than water-lain deposits.

Supplementary Note 5: Rock magnetic measurements

Normalized χ-T heating curves for selected samples are shown in Figure 5. Heating curves for the upper part of the studied sequence (Supplementary Figure 5a-c) generally increase to a hump at 400 ℃ before declining to 500 ℃, and then increase to a Hopkinson peak for magnetite at ~580 ℃ before decaying sharply and then more gradually from 600 ℃ to 700 ℃. These features are associated with the coexistence of maghemite,

magnetite, and hematite, respectively. For samples from the lower part of the section (Supplementary Figure 5d-f), similar features are observed during heating, although the hump at 400 ℃ associated with maghemite is larger, and the χ-T cooling curves are relatively flat above 200 ℃, which indicates that magnetite particles occur in the multi-domain state. The cooling curves have lower χ values than the heating curves, which is usually caused by conversion of fine-grained maghemite to hematite at elevated temperatures[27].

Overall, the magnetic mineral assemblage for the studied sequence is relatively uniform with the coexistence of coarse-grained magnetite and hematite. The major difference is that the lower section (older than ~47 Ma) contains more fine-grained maghemite particles, and hematite becomes gradually the more dominant magnetic mineral from the lower to the upper part of the section.

Supplementary Note 6: Palaeomagnetic results

Demagnetization results were evaluated using orthogonal vector component diagrams[28] (Supplementary Figure 6). A secondary natural remanent magnetization (NRM) component was removed by thermal demagnetization treatment up to about 300 ℃. Characteristic remanent magnetization (ChRM) directions were calculated using least-squares principal component analysis[24] with the maximum angular deviation (MAD) determined from a minimum of four demagnetization measurements for the high-temperature NRM component. Virtual geomagnetic pole (VGP) latitudes were calculated from the ChRM directions. Each major magnetic polarity zone for the studied

sedimentary succession is defined by several samples with the same polarity.

Supplementary Note 7: NRM recording mechanism in aeolian deposits

Previous studies have shown that aeolian sediments on the Chinese Loess Plateau (CLP) contain coarse-grained (vortex state/multi-domain) low-Ti magnetite and hematite, which record a combined depositional remanent magnetization (DRM) and post-depositional remanent magnetization (pDRM) with maximum unblocking temperatures close to the Curie/Néel temperatures of magnetite (580 ℃) and hematite (670 ℃), respectively[29]. Pedogenic processes produce maghemite and hematite nanoparticles. Pedogenic hematite will carry a chemical remanent magnetization (CRM) that can overprint the high-temperature palaeomagnetic components to varying degrees above 600 ℃. Generally, red bed remagnetization can be attributed to two processes: CRM acquisition in hematite and post-depositional realignment of coarse magnetite in sand layers. There is general consensus that the A- and B-horizons of soils will be enhanced magnetically by neoformation of maghemite and hematite nanoparticles through transformation of ferrihydrite[30]. Thus, CRM acquisition will shift the palaeomagnetic record downward to some depth, in a similar manner to pDRM lock-in. Such a CRM should still be useful for magnetostratigraphy because pedogenic hematite formation is rapid and occurs before deeper burial (as is to be expected for soil-forming processes, e.g., the Holocene soil on the CLP). Therefore, this type of CRM acquisition should not affect significantly the overall polarity pattern recorded by aeolian deposits.

The CRM issue has been well considered for the Chinese loess since the initial palaeomagnetic studies by Heller and Liu[31]. There is now consensus that CRM does not affect greatly the magnetostratigraphy of Chinese loess. Magnetostratigraphy has been the most robust tool for dating the long Chinese loess and red clay sequences since the late Miocene[31], the early Miocene (< ~22 Ma)[8], and the late Oligocene[15]. They have similar depositional settings; therefore, such a rationale can be extended reasonably to other aeolian material such as the red beds in this study.

Concerning the second remagnetization mechanism, water is important for pDRM acquisition in aeolian deposits, but remanence acquisition differs from that in lake or marine sediments, where magnetization lock-in is driven largely by gradual compaction. With high enough sedimentary water content during early burial, coarse particles can rotate physically and cause realignment of an original DRM[32,33]. However, this occurs only in coarse layers (such as L9 in the Chinese loess), where water can easily seep down to affect particle alignment at deeper levels[34].

Preservation of an original magnetization in the studied palaeomagnetic record is supported by a positive reversal test. With CRM overprinting, both normal and reversed polarity palaeomagnetic directions are likely to be shifted from the original directions, and would no longer be antipodal[35]. For our data, the angle between mean normal and reversed polarity directions is 12.9°. The critical angle (γ_c) between normal and reversed polarity directions for the studied site is 14.9°. Thus, the reversal test passes with a C classification of McFadden and McElhinny[35] at the 95% confidence level

(Supplementary Figure 7). This indicates that the overall magnetostratigraphy has not been contaminated significantly by remagnetization.

Supplementary Note 8: Palaeomagnetic excursions

Earth’s magnetic field varies on different time scales, with different types of behaviour observed, including secular variation, palaeomagnetic excursions, and polarity reversals. A geomagnetic excursion is defined as a deviation of the VGP for several thousand years by more than 40–45 ° from the geographic pole[36] or as a deviation of VGPs away from the normal range of geomagnetic secular variation[37]. Previous studies have shown that excursions occur widely in late Quaternary palaeomagnetic records[38, 39]. Geomagnetic excursions have also been recorded in Chinese loess sequences[40]. In the GPTS obtained from marine magnetic anomaly profiles, Cande and Kent[41,42] reported short-period anomalies with ambiguous origin, which are referred to as ‘tiny wiggles’. Three mechanisms have been proposed for the origin of these tiny wiggles[41,43]. They are interpreted to represent either: (1) large-scale palaeointensity variations without accompanying directional excursions, (2) geomagnetic excursions, or (3) short polarity subchrons. Based on studies of the Brunhes Chron, most major intensity variations are accompanied by directional excursions[38,39], so even for the most conservative of the three mechanisms proposed, it is reasonable to expect tiny wiggles to at least sometimes coincide with a geomagnetic excursion. Tiny wiggles occur frequently in chrons C20n, 20r, C21n, C21r, and C22r[41], which provides a basis to expect short-duration features such as excursions to have occurred through the time period represented by the studied

sedimentary sequence (see Figure 3 in the main text). Also, it is reasonable to expect excursions to be present because the geomagnetic field should have varied in a similar manner in deeper time as in more recent time.

Previous studies have shown that aeolian sediments can record faithfully palaeomagnetic polarity subchrons, but that they are less efficient recorders of detailed transitional field behaviour associated with short-lived excursions or polarity reversals over periods of several thousand years[44]. This is mainly thought to be due to the poor alignment efficiency of magnetic particles during periods with weak palaeomagnetic field intensities during these events. Therefore, large discrepancies exist for palaeomagnetic records of such events for sister samples from the same stratigraphic level. Rather than this being a weakness, we use this possibility to assess the underlying mechanism for short-lived palaeomagnetic directional anomalies.

Supplementary Note 9: Magnetostratigraphy

Stratigraphic plots of the frequency dependence of susceptibility (χ_{fd}) and palaeomagnetic results for the studied red clay sequence are shown in Supplementary Figures. 8 and 9. Discovery of the *Yuomys* rodent fossil indicates unambiguously that the studied red clay sequence belongs to the Sharamurunian period, which spans the late Middle Eocene age interval[45,46].

To further determine the recording fidelity of palaeomagnetic events with the shortest durations from the studied sequence, we remeasured 62 parallel samples. For long-term subchrons, both sets of samples yield consistent results. In contrast, the short-lived events

at ~32-33 m and 70-72 m yield inconsistent results, which strongly indicate that these are excursional intervals. The two sample sets yield consistent results over four intervals at depths of ~43 m, ~61 m, 62 m, and ~63 m. It is difficult to judge the origin (excursion or short subchron) of these short-term polarity anomalies. Therefore, we present two alternative correlations (Supplementary Figures 6 and 7) between our palaeomagnetic polarity results and the geomagnetic polarity timescale (GPTS)[47] that are consistent with the Eocene age indication from the presence of *Yuomys* fossils (Supplementary Figure 9).

For the first interpretation (Supplementary Figure 8), the short-lived directional anomalies at ~43 m, 46 m, 61 m, and 63 m are interpreted to represent excursions rather than subchrons (Supplementary Figure 9). In this case, the magnetic polarity pattern for the studied sediments is constructed using the thicker polarity zones that are defined by larger numbers of samples that record the same polarity. For the second interpretation, these short-lived directional anomalies are assigned to subchrons. In the first correlation option, the age of the sequence is assigned to between ~39 and ~51 Ma, while in the second correlation option the sequence is dated to between ~39 and ~54 Ma. For the first option, the sediment accumulation rate is uniform (~10 m/Ma) (Supplementary Figure 10a). In contrast, the second correlation yields large sediment accumulation rate fluctuations that range between ~1 and ~78 m/Ma, and an inconsistent pattern between χ_{fd} and global marine $\delta^{18}O$ variations (Supplementary Figure 11**)**. The more consistent sedimentation rates, which are typical of Asian aeolian deposits[29], and the striking correlation between χ_{fd} and global marine $\delta^{18}O$ variations (main text Figure 3d, e) that indicates recording of global climate variations in the studied red clay sequence, favours

strongly the first correlation option. Nevertheless, both correlations support the conclusion that the studied red clay sequence has a basal age >51 Ma.

For comparison, we provide a third correlation by only matching the paleomagnetic polarity pattern to the GPTS (Figure 12a). On the basis of this correlation, the sequence is dated to between 39 and 35 Ma. *Yuomys altunensis* has an age of ~35.8 Ma, which is associated with the Sharamurunian stage in the Asian Land Mammal Age timescale. This youngest age model yields large sedimentation rate fluctuations (Supplementary Figure 12b) and is inconsistent with the age of *Yuomys altunensis*. Based on these factors, we dismiss this third age correlation option.

Supplementary Note 10: Identification of a *Yuomys* fossil from the Altun region, China[45]

Designation: *Yuomys* ***altunensis***

Material: Several upper jaw teeth (P^4 root and $M^{1\text{-}3}$, $M^{1\text{-}2}$, $M^{2\text{-}3}$, and M^2).

Location and Stratigraphy: East of Caihong ditch, lower Xishungou Formation (second layer), Altun region.

Description: Individual sizes are relatively large. The upper jaw teeth are characterized by moderate lingual crown and hill ridge. P^4 is greater than M^1. The posterior ridge of the upper molars is oblique with medial forward extension, pointing to the protocone. The metaconule is clear, and is not swollen. The hypercone of $M^{1\text{-}2}$ is similar in size to the protocone, and is located behind it.

Age: All known *Yuomys* ages are from the Middle Eocene. The stratigraphic age of *Yuomy*s *altunensis* is dated to the late-Middle Eocene (Sharamurunian Asian Land Mammal Age) in the Altun region. Species of *Yuomys* with individual sizes from small to large have an evolutionary trend. The large tooth size of the Altun species is similar to *Yuomys cavioides* and *Y. huangzhuangensis*, which have a middle Eocene age.

Surveyor: Banyue Wang

Identification date: 2016-6-13

Supplementary **Table 1. Description of the geological profile.**

**Units**	**Thickness (m)**	**Bottom Age (Ma)**	**Features**	**Red clay/carbonate couplets**
Unit-1	1.2	40.01	Reddish-brown clay, massive structure, with abundant ginger-like and branch-like carbonate nodules.	1
Unit-2	14.2	41.24	Yellowish-brown clay, massive structure, interbedded with greyish-green carbonate nodule horizons.	2–7
Unit-3	4.6	41.62	Massive brownish clay with a few grey spherical carbonate nodules (2–5 mm).*Yuomys altunensis* **fossils** are found in the upper part of this unit.	8–9
Unit-4	3.2	41.85	Red and purplish-red clay, characterized by ginger-like and branch-like carbonate nodules.	10
Unit-5	1.8	41.97	Yellowish-brown clay with massive structure, containing fibre-like carbonate nodules.	11–12
Unit-6	3.6	42.23	Massive brownish clay, interbedded with greyish-green carbonate nodule layers.	13–16
Unit-7	9.0	43.22	Purplish-red clay (characterized by ginger-like and branch-like carbonate nodule in the clay), interbedded with greyish-yellow and greyish-green carbonate nodule layers.	17–21
Unit-8	2.4	43.55	Yellowish-brown clay, interbedded with greyish-yellow carbonate nodule layers.	22–23
Unit-9	9.6	45.38	Red and purplish-red clay, interbedded with grey carbonate nodule horizons. The clay contains fibre-like and irregular carbonate nodules.	24–26
Unit-10	5.8	46.20	Dominated by grey and greyish-green carbonate nodule layers, interbedded with purplish-red clay.	27–28
Unit-11	5.4	46.83	Brownish-red and purplish-red clay (containing many spherical 10–30 mm carbonate nodules), interbedded with yellowish-green carbonate nodule layers.	29–32
Unit-12	5.8	47.50	Brownish clay, interbedded with greyish-green carbonate nodule layers. The clay contains spherical carbonate nodules (2–5 mm).	33
Unit-13	1.0	47.61	Reddish-brown clay, interbedded with greyish-green carbonate nodule horizons.	34
Unit-14	4.2	48.07	Massive brownish clay containing abundant spherical carbonate nodules (10–20 mm).	35
Unit-15	2.4	48.33	Brownish-red clay, interbedded with greyish-green carbonate nodule horizons. The clay contains spherical carbonate nodules.	36
Unit-16	1.8	48.52	Brownish clay with larger, irregular carbonate nodules.	37
Unit-17	2.8	49.12	Reddish-brown clay, with massive structure, interbedded with three greyish-green carbonate nodule horizons.	38
Unit-18	8.2	50.29	Brownish clay interbedded with greyish-green carbonate nodule layers.	39–40
Unit-19	8.8	>51.00	Reworked brownish clay with gravels (2–5 mm, angular, poorly sorted), massive structure. The clay contains spherical carbonate nodules (5–10 mm).	

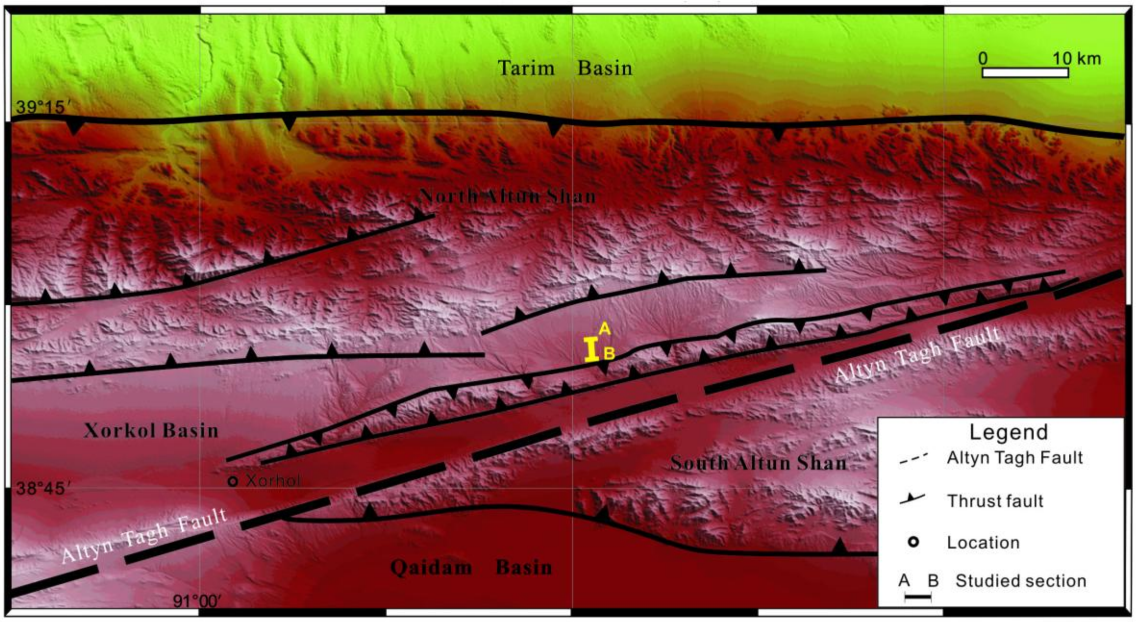

Supplementary Figure 1. Close-up topographic map of the study location and surrounding region. The digital elevation data are from http://www.gscloud.cn/.

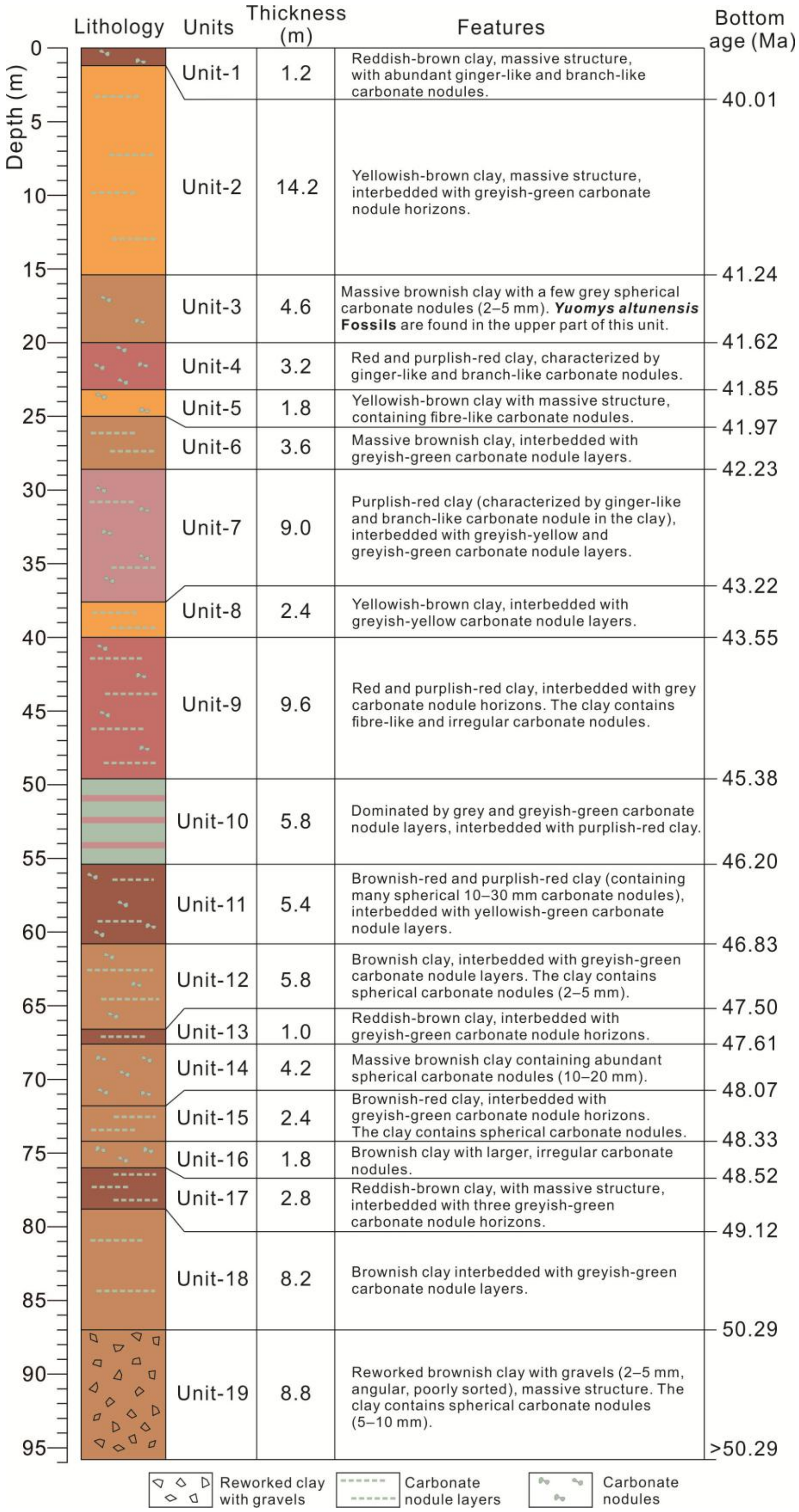

Supplementary Figure 2. Lithology of the studied profile.

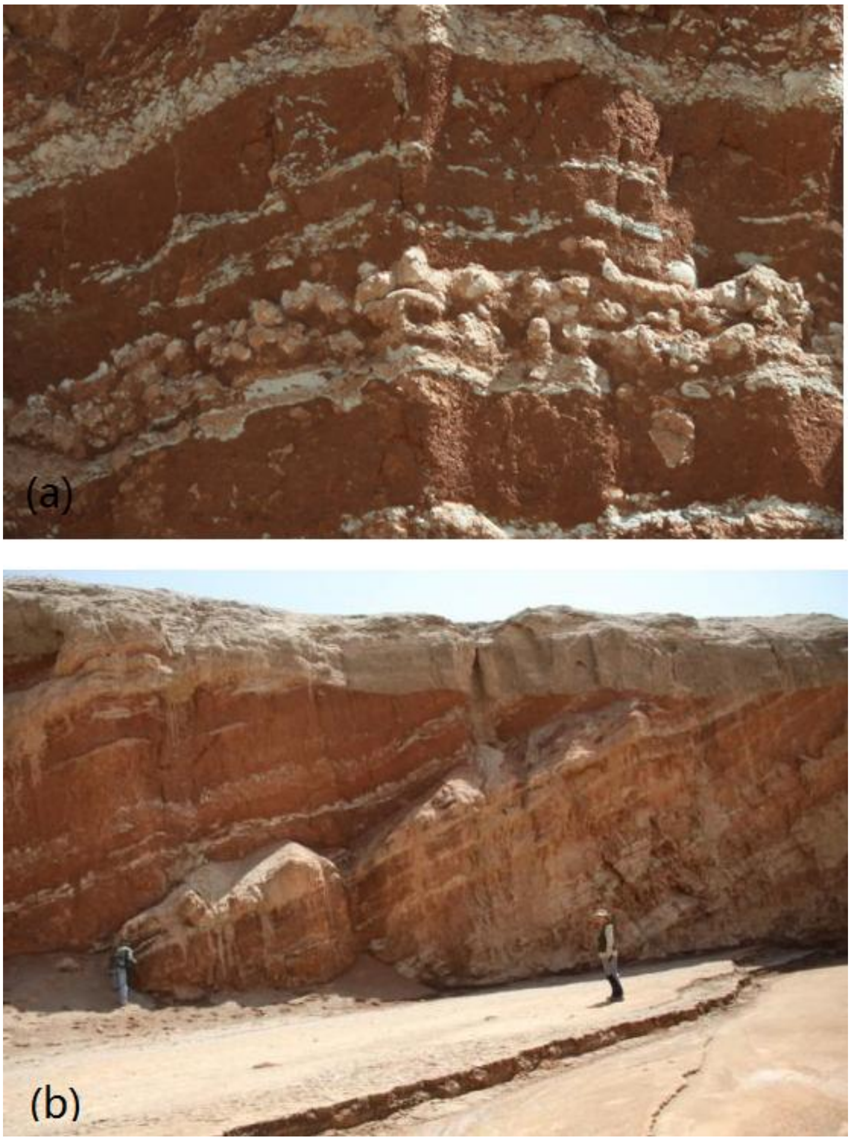

Supplementary Figure 3. Field photographs of “pseudobedding” in the studied red clay sequence. (a) Close-up and (b) distant views.

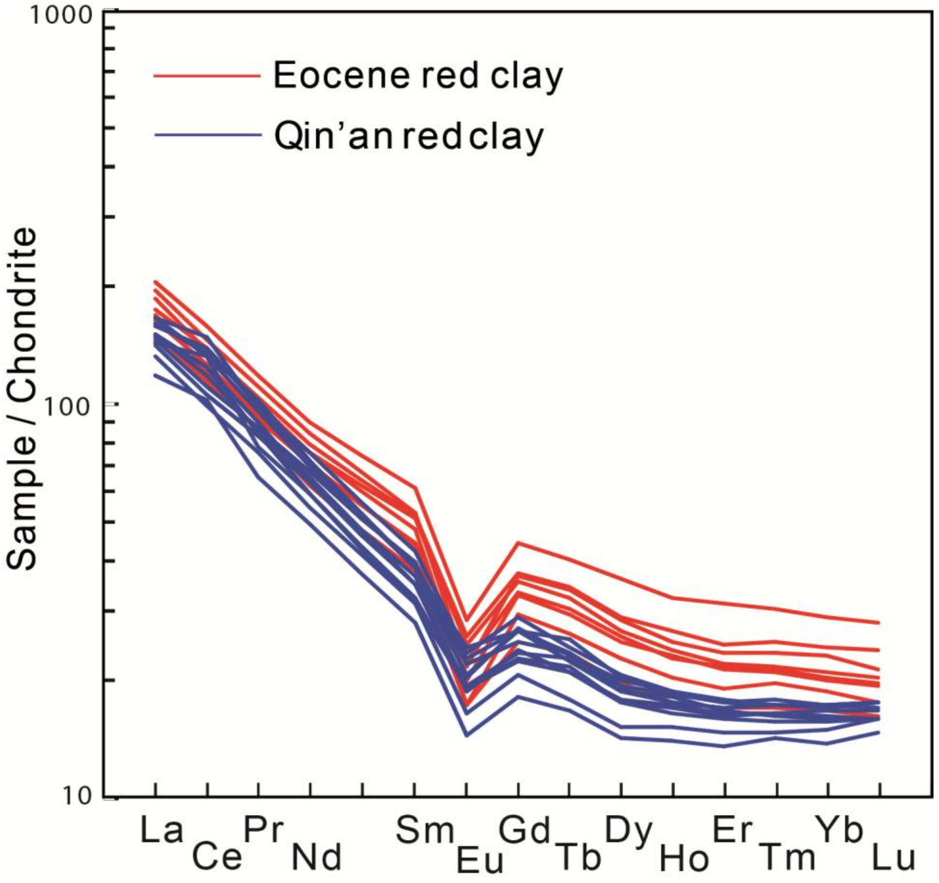

Supplementary Figure 4. Rare Earth element distribution patterns for the studied red clay from Altun Shan compared with red clay from Qin'an (ref. 12).

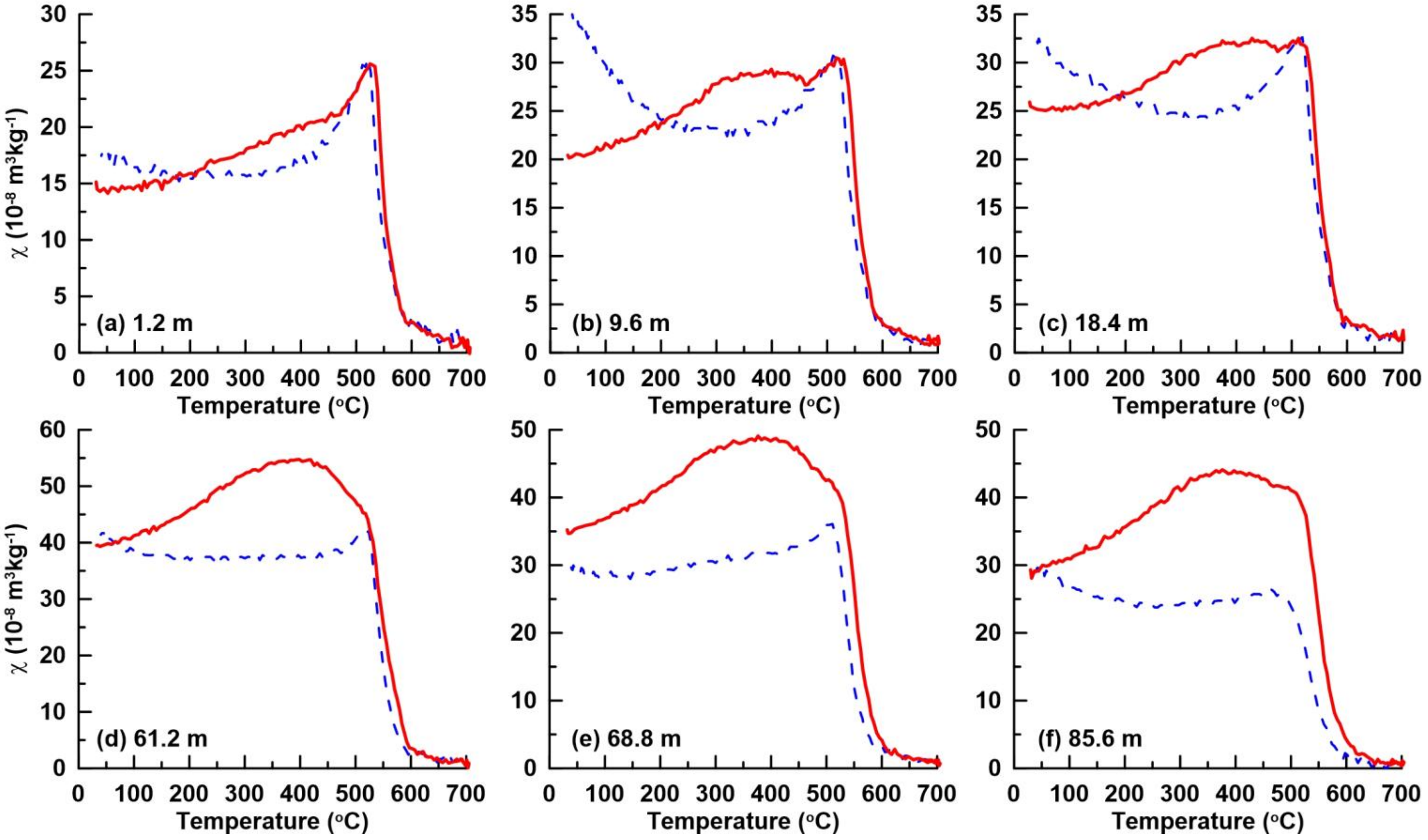

Supplementary Figure 5. High-temperature magnetic susceptibility (χ–*T*) curves (red = heating; blue = cooling) for representative samples, in metres from the top of the studied section. All samples contain a mixture of magnetite and hematite.

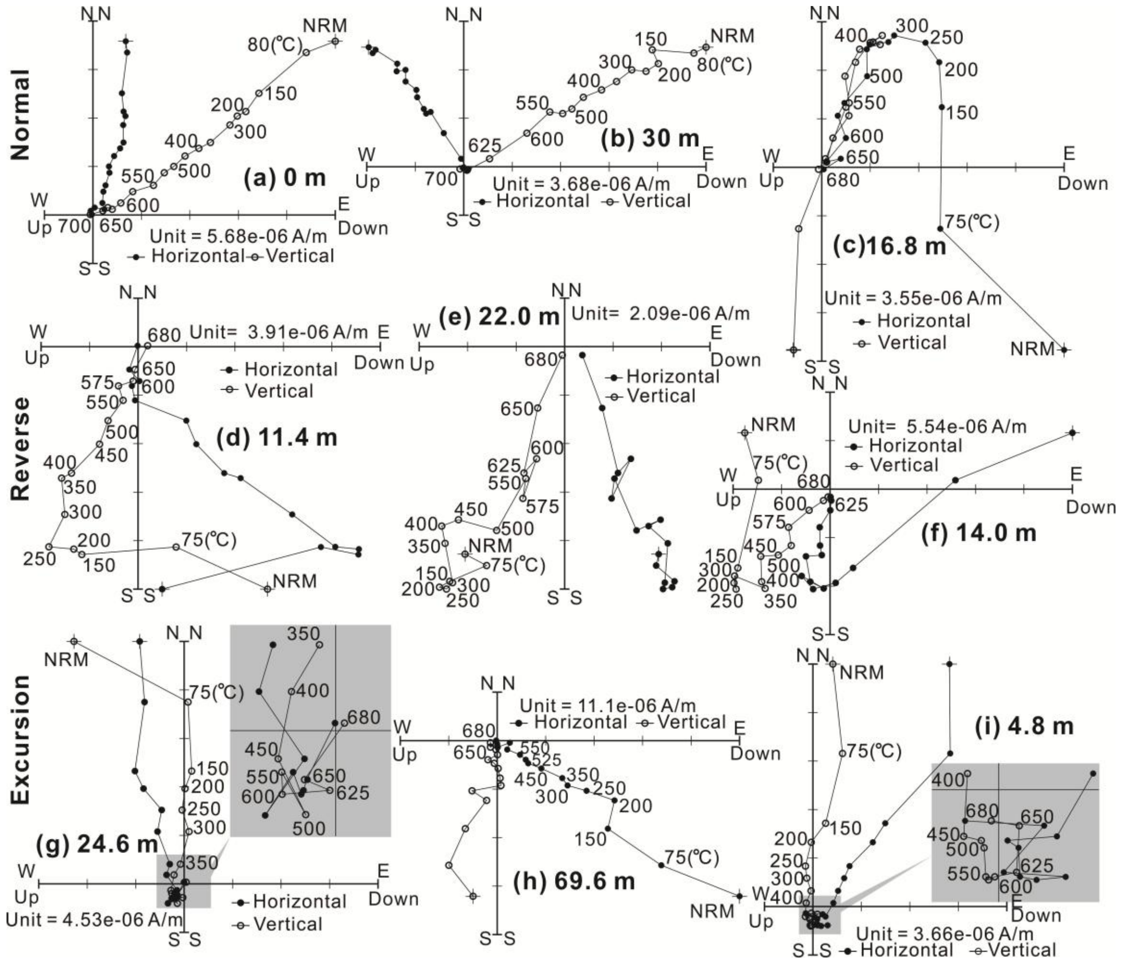

Supplementary Figure 6. Representative orthogonal vector projections of stepwise thermal demagnetization data for samples from the studied section, in metres below the top of the section. Open and closed circles indicate magnetization vectors projected onto the vertical and horizontal planes, respectively.

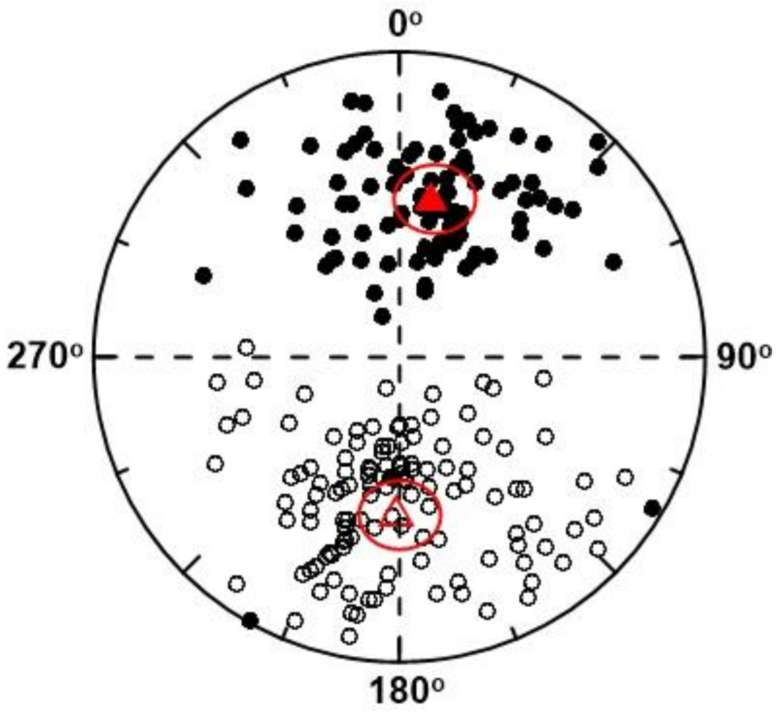

Supplementary Figure 7. Stereographic projection of palaeomagnetic ChRM directions after analysis of stepwise demagnetization data as presented in Supplementary Figure 2. Solid and open circles indicate projections onto the upper and lower hemispheres, respectively. Solid and open triangles indicate mean directions of the normal and reversed polarity directions, respectively. Red ellipses represent the 95% confidence intervals about the respective means.

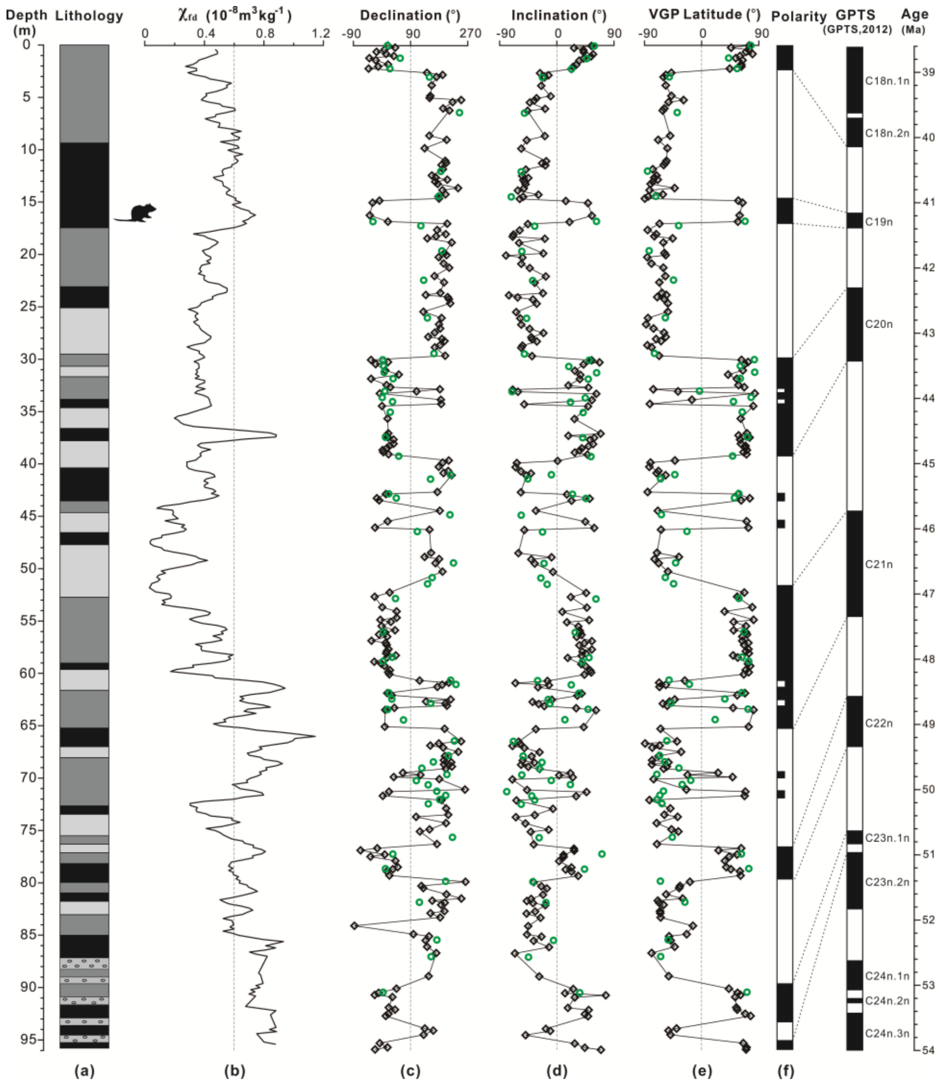

Supplementary Figure 8. The first correlation option between virtual geomagnetic poles (VGPs) for the studied red clay sequence and the GPTS. (a) Lithology, (b) frequency-dependence of magnetic susceptibility (χ_{fd}), (c) declination, (d) inclination, (e) VGP latitude, and (f) magnetic polarity stratigraphy of the studied Xishuigou section. Green open circles indicate results from parallel samples. Dashed lines indicate the correlation between the interpreted polarity and the GPTS by assigning the short-lived palaeomagnetic anomalies as excursions. Polarity: black = normal, white = reversed; short black and white bars within the major subchrons indicate the positions of interpreted geomagnetic excursions.

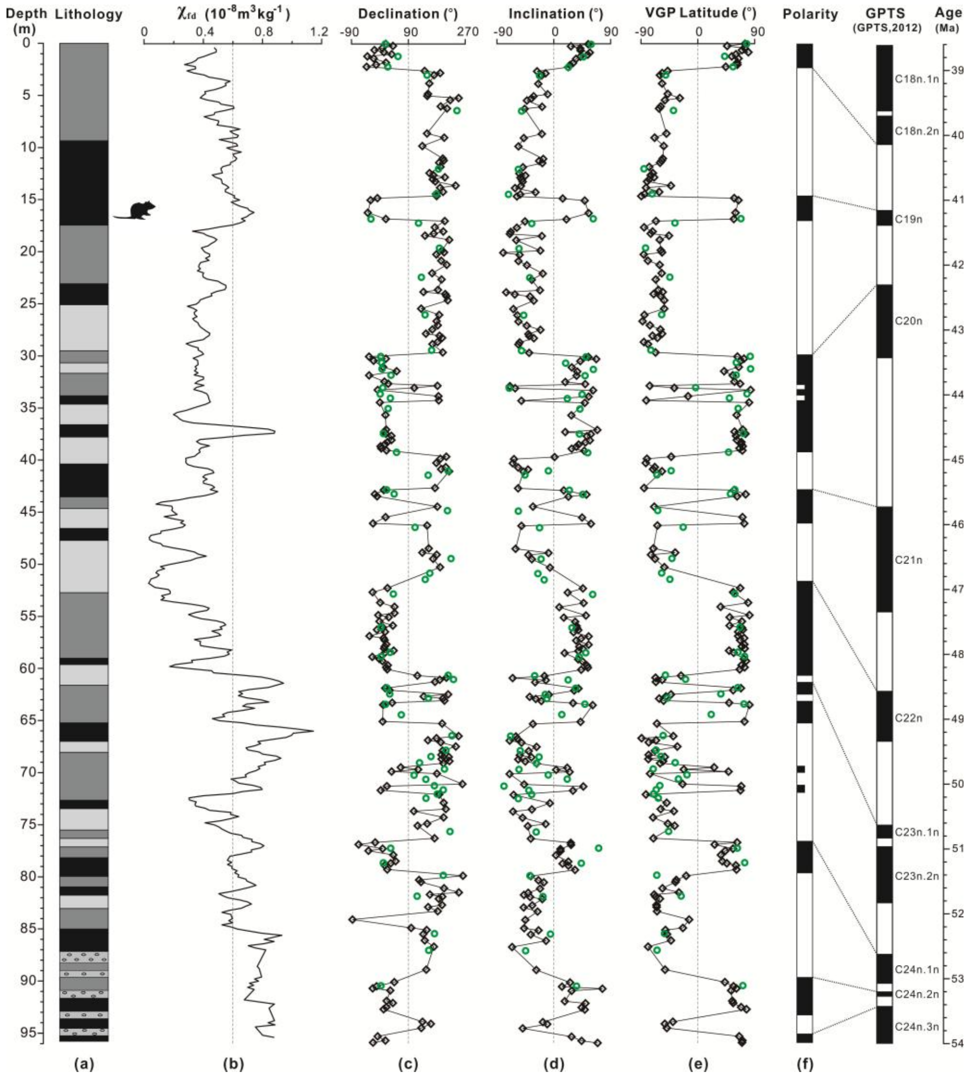

Supplementary Figure 9. The second correlation option between VGPs for the studied red clay sequence and the GPTS. (a) Lithology, (b) frequency-dependence of magnetic susceptibility (χ_{fd}), (c) declination, (d) inclination, (e) VGP latitude, and (f) magnetic polarity stratigraphy of the studied Xishuigou section. Green open circles indicate results from parallel samples. Dashed lines indicate the correlation between the interpreted polarity and GPTS by assigning the short-lived palaeomagnetic anomalies as subchrons. Polarity: black = normal, white = reversed; short black and white bars within the major subchrons indicate the positions of interpreted geomagnetic excursions.

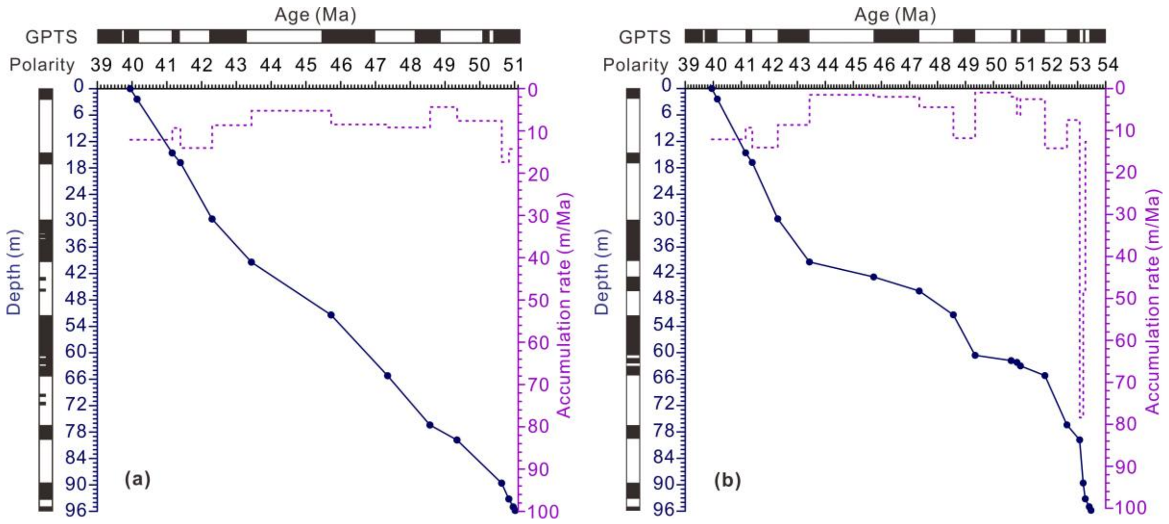

Supplementary Figure 10. Age models (blue) and sediment accumulation rates (purple) for two alternative correlations between palaeomagnetic results for the studied red clay sequence and the GPTS as shown in Supplementary Figures 4 and 5. In (b), the second correlation option, the sedimentation rate undergoes large fluctuations.

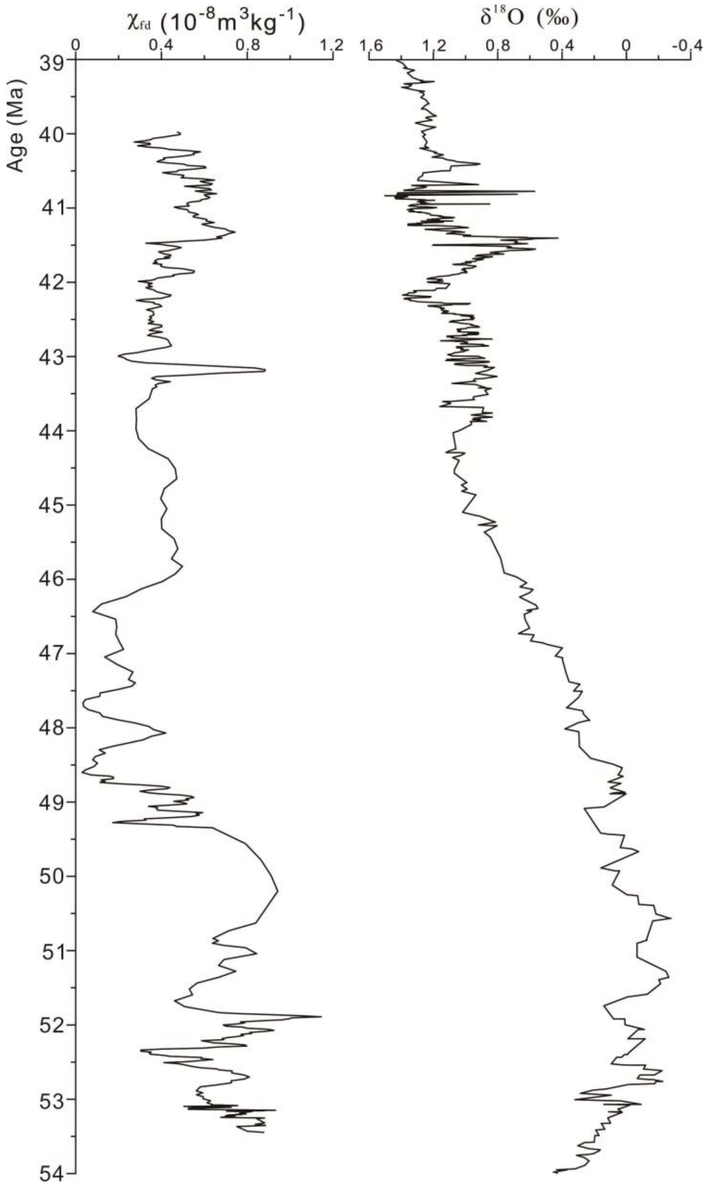

Supplementary Figure 11. Age plots of χ_{fd} and δ^{18}O of ref. 48. The age model is from the second correlation option between VGPs for the studied red clay sequence and the GPTS (Supplementary Figure S6b). Compared to the excellent correspondence between χ_{fd} and δ^{18}O from the first correlation option (main text Figure 3d, e), these parameters do not correlate well, thus, the corresponding age model (second correlation opinion) is not favoured.

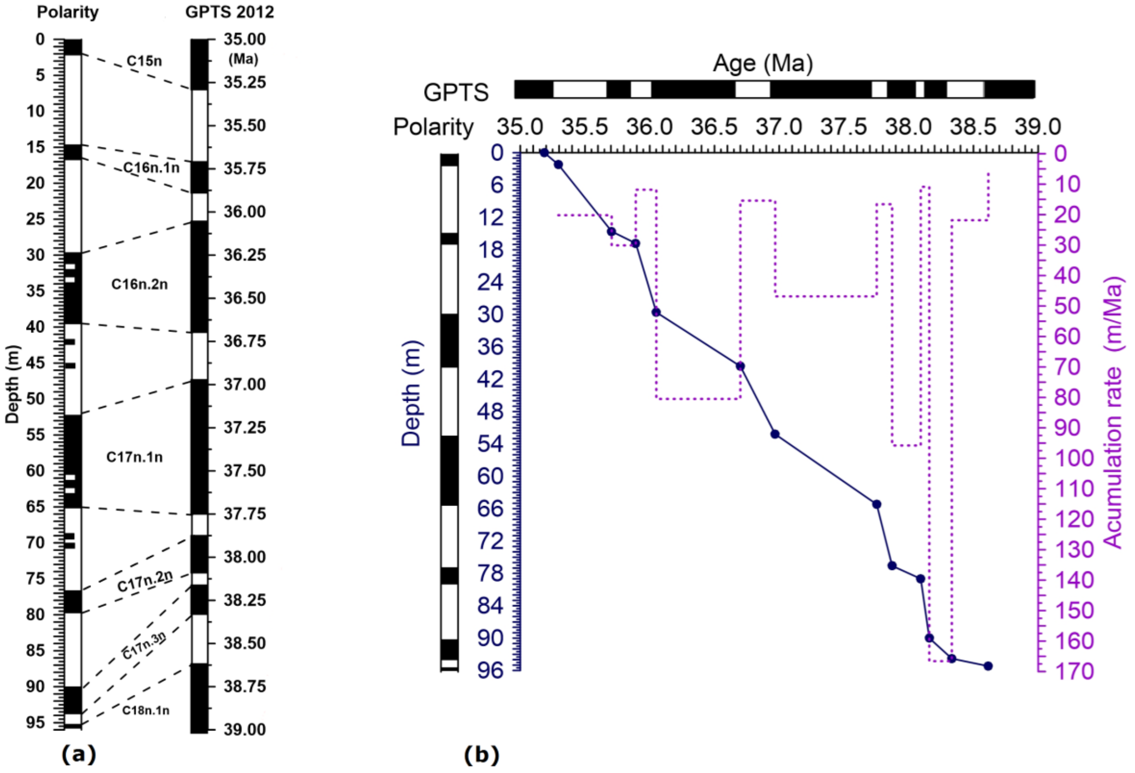

Supplementary Figure 12. (a) Depth plot of paleomagnetic results for the studied red clay sequence and options for correlation with the geomagnetic polarity timescale (GPTS). Polarity: black = normal, white = reverse; short black and white bars within the major subchrons indicate the positions of interpreted geomagnetic excursions. (b) Age models (blue) and sediment accumulation rates (purple) for correlation between paleomagnetic results for the studied red clay sequence and the GPTS as shown in (a). The sedimentation rate undergoes large fluctuations.

Supplementary References

1. The Geological Survey Team of the Xinjiang Uygur Autonomous Region. Regional Map and Report of the Geology and Mineral Resources for the Suoerkuli sheet at the scale of 1:200,000. Urumqi: The Geological Survey Team of the Xinjiang Uygur Autonomous Region (in Chinese) (1981).

2. Yue, Y. J. *et al*. Slowing extrusion tectonics: lowered estimate of post-Early Miocene slip rate for the Altyn Tagh fault. *Earth Planet. Sci. Lett.* **228**, 311–323 (2004).

3. Yue, Y. J. *et al*. Detrital zircon provenance evidence for large-scale extrusion along the Altyn Tagh fault. *Tectonophysics* **406**, 165–178 (2005).

4. Li, J. X. *et al*. Intensified aridity of the Asian interior recorded by the magnetism of red clay in Altun Shan, NE Tibetan Plateau. *Palaeogeogr. Palaeoclimatol. Palaeoecol.* **411**, 30–41 (2014).

5. Heller, F. & Evans, M. E. Loess magnetism. *Rev. Geophys.* **33**, 211–240 (1995).

6. Taylor, S. R. *et al.* Geochemistry of loess, continental crustal composition and crustal model ages. *Geochim. Cosmochim. Acta* **47**, 1897–1905 (1983).

7. Gallet, S. *et al*. Loess geochemistry and its implications for particle origin and composition of the upper continental crust. *Earth Planet. Sci. Lett.* **156**, 157–172 (1998).

8. Guo, Z. *et al*. Onset of Asian desertification by 22 Myr ago inferred from loess deposits in China. *Nature* **416**, 159–163 (2002).

9. Ding, Z. *et al.* Iron geochemistry of loess and red clay deposits in the Chinese Loess Plateau and implications for long-term Asian monsoon evolution in the last 7.0 Ma. *Earth Planet. Sci. Lett.* **185**, 99–109 (2001).

10. Sun, J. & Windley, B. F. Onset of aridification by 34 Ma across the Eocene-Oligocene transition in Central Asia. *Geology* **43**, 1015–1018 (2015).

11. Sun, J. *et al*. Late Oligocene–Miocene mid-latitude aridification and wind patterns in the Asian interior. *Geology* **38**, 515–518 (2010).

12. Liang, M. *et al*. Geochemical characteristics of the Miocene eolian deposits in China: their provenance and climate implications. *Geochem. Geophys. Geosyst.* **10**, Q04004, doi:10.1029/2008GC002331 (2009).

13. Taylor, S. R. & McLennan, S. M. *The Continental Crust: Its Composition and Evolution.* Blackwell, Oxford (1985).

14. Pye, K. & Tsoar, H. *Aeolian Sand and Sand Dunes.* Springer, Berlin (2009).

15. Qiang, X. *et al.* New eolian red clay sequence on the western Chinese Loess Plateau linked to onset of Asian desertification about 25 Ma ago. *Sci. China* **54**, 136–144 (2011).

16. An, Z. *et al*. Evolution of Asian monsoons and phased uplift of the Himalaya–Tibetan plateau since Late Miocene times. *Nature* **411**, 62–66 (2001).

17. Zhang, Y. F. *et al*. Magnetic fabric characteristics of sand-dune sediments and its paleowind field in the middle reaches of Yangtze River. *Chin. J. Geophys.* **52**, 95–101 (2009).

18. Pye, K. The nature, origin and accumulation of loess. *Quat. Sci. Rev.* **14**, 653–667 (1995).

19. Liu, T. S. *Loess and the Environment*. China Ocean Press, Beijing (1985).

20. Rea, D. K. *et al*. Late Cenozoic eolian deposition in the North Pacific: Asian drying, Tibetan uplift, and cooling of the northern hemisphere. *Paleoceanography* **13**, 215–224 (1998).

21. Pye, K. & Zhou, L. P. Late Pleistocene and Holocene aeolian dust deposition in North China and the Northwest Pacific Ocean. *Palaeogeogr. Palaeoclimatol. Palaeoecol.* **73**, 11–23 (1989).

22. Wang, P. *et al*. Saltation and suspension of wind-blown particle movement. *Sci. China* **51**, 1586–1596 (2008).

23. McTainsh, G. & Strong, C. The role of aeolian dust in ecosystems. *Geomorphology* **89**, 39–54 (2007).

24. Kirschvink, J. L. The least-squares line and plane and the analysis of palaeomagnetic data. *Geophys. J. R. Astron. Soc.* **62**, 699–718 (1980).

25. Pavelić, D. *et al*. Early Miocene European loess: a new record of aridity in southern Europe. *Geol. Soc. Am. Bull.* **128**, 110–121 (2016).

26. Licht, A. *et al.* Asian monsoons in a late Eocene greenhouse world. *Nature* **513**, 501–506 (2014).

27. Liu, Q. S. *et al*. Temperature dependence of magnetic susceptibility in an argon environment: implications for pedogenesis of Chinese loess/palaeosols. *Geophys. J. Int.* **161**, 102–112 (2005).

28. Zijderveld, J. D. A. AC demagnetization of rocks: analysis of results, *Methods in Palaeomagnetism*. Collinson, D. W. *et al*. (eds), 254–286. Elsevier, New York (1967).

29. Liu, Q. S. *et al*. Magnetostratigraphy of Chinese loess–paleosol sequences. *Earth-Sci. Rev.* **150**, 139–167 (2015).

30. Liu, Q. S. *et al*. Environmental magnetism: principles and applications. *Rev. Geophys.* **50**, RG4002, doi:10.1029/2012RG000393 (2012).

31. Heller, F. & Liu, T. S. Magnetostratigraphical dating of loess deposits in China. *Nature* **300**, 431–433 (1982).

32. Zhao, X. & Roberts, A. P. How does Chinese loess become magnetized? *Earth Planet. Sci. Lett.* **292**, 112–122 (2010).

33. Wang, R. & Løvlie, R. Subaerial and subaqueous deposition of loess: experimental

assessment of detrital remanent magnetization in Chinese loess. *Earth Planet. Sci. Lett.* **298**, 394–404 (2010).

34. Jin, C. & Liu, Q. Remagnetization mechanism and a new age model for L9 in Chinese loess. *Phys. Earth Planet. Inter.* **187**, 261–275 (2011).

35. McFadden, P. L. & McElhinny, M. W. Classification of the reversal test in palaeomagnetism. *Geophys. J. Int.* **103**, 725–729 (1990).

36. Merrill, R. T. & McFadden, P. L. Geomagnetic field stability: reversal events and excursions. *Earth Planet. Sci. Lett.* **121**, 57–69 (1994).

37. Vandamme, D. A new method to determine paleosecular variation. *Phys. Earth Planet. Inter.* **85**, 131–142 (1994).

38. Laj, C. & Channell, J. E. T. Geomagnetic excursions. *Treatise on Geophysics* **5**, 373–416 (2007).

39. Roberts, A. P. Geomagnetic excursions: knowns and unknowns. *Geophys. Res. Lett.* **35**, L17307, doi:10.1029/2008GL034719 (2008).

40. Pan, Y. *et al*. Can relative paleointensities be determined from the normalized magnetization of the wind-blown loess of China? *J. Geophys. Res.* **106**, 19221–19232 (2001).

41. Cande, S. C. & Kent, D. V. Ultrahigh resolution marine magnetic anomaly profiles: a record of continuous paleointensity variations? *J. Geophys. Res.* **97**, 15,075–15,083 (1992).

42. Cande, S. C. & Kent, D. V. A new geomagnetic polarity time scale for the Late Cretaceous and Cenozoic. *J. Geophys. Res.* **97**, 13,917–13,951 (1992).

43. Roberts, A. P. & Lewin-Harris, J. C. Marine magnetic anomalies: evidence that 'tiny wiggles' represent short-period geomagnetic polarity intervals. *Earth Planet. Sci. Lett.* **183**, 375–388 (2000).

44. Jin, C. S. & Liu, Q. S. Revisiting the stratigraphic position of the Matuyama-Brunhes geomagnetic polarity boundary in Chinese loess. *Palaeogeogr. Palaeoclimatol. Palaeoecol*. **299**, 309–317 (2011).

45. Wang B. Discovery of *Yuomys* from Altun Shan, Xinjiang, China. *Vertebrata Palasiatica* **55**, 227–232 (2017).

46. Wang, Y. *et al*. Major events of Paleogene mammal radiation in China. *Geol. J.* **42**, 415–430 (2007).

47. Gradstein, F. M. *et al*. *The Geologic Time Scale 2012*. Elsevier (2012).

48. Zachos, J. C. *et al*. An early Cenozoic perspective on greenhouse warming and carbon-cycle dynamics. *Nature* **451**, 279–283 (2008).
